# Supplementary material for: BLEND: a fast, memory-efficient and accurate mechanism to find fuzzy seed matches in genome analysis
Source: NAR Genom Bioinform. 2023 Jan 20;5(1):lqad004. doi: 10.1093/nargab/lqad004 (PMC9853099; doi:10.1093/nargab/lqad004)
Supplement: lqad004_Supplemental_File [file lqad004_supplemental_file.pdf]

Supplementary Material for BLEND: A Fast, Memory-Efficient, and Accurate Mechanism to Find Fuzzy Seed Matches in Genome Analysis

S1. Statistics of Fuzzy Seed Matching

S1.1. Finding Fuzzy Matches Between Minimizers

Supplementary Table S1 shows the overall statistics of the fuzzy seed matching we explain in the *Empirical Analysis of Fuzzy Seed Matching* section. We find minimizers using 1) the low collision hash function that minimap2 uses (i.e., `hash64`) and 2) the SimHash technique (1, 2) we use in BLEND. For BLEND, we use the `BLEND-I` technique to directly compare the minimizers found using BLEND and minimap2. We keep the seed length constant, 16. For BLEND, we use various numbers of immediately overlapping k-mers that BLEND extracts from seed sequences (i.e., *neighbors*), as explained in the *Sequence to Set Conversion* section. To keep the seed length ($|S|$) constant with a varying number of neighbors (n), we calculate the k-mer length (k) we extract from seeds as follows: $|S| = n+k-1$ where $|S|$ and n are known. For each tool and configuration, we report the overall number of minimizers we find, the number of minimizer pairs that generate the same hash value (i.e., *collision*), the ratio of collisions to all minimizers, and the average edit distance between the minimizer pairs that have the same hash value. We make our resulting dataset that includes the statistics shown in Figure 7 and Supplementary Table S1 available at Zenodo[1].

Table S1. Fuzzy seed matching statistics of minimizer seeds that we find using minimap2 and BLEND. The number of overlapping k-mers that BLEND extracts from seed sequences (i.e., neighbors or n) are annotated as BLEND-n

Tool	Number of Minimizers	Number of Collisions	Collision/Minimizer Ratio	Avg. Edit Distance Between Minimizers With Collision
minimap2	903,043	15,306	0.016949	9.327061
BLEND-3	1,014,173	18,224	0.017969	9.393437
BLEND-5	1,090,468	20,659	0.018945	9.213660
BLEND-7	1,140,254	23,591	0.020689	8.874698
BLEND-9	1,173,198	28,411	0.024217	8.495301
BLEND-11	1,186,687	35,500	0.029915	8.067549
BLEND-13	1,197,966	72,078	0.060167	8.075918

S1.2. Finding Fuzzy Matches Between Similar Sequences

Our goal is to find the non-identical k-mers with the same hash value between similar sequences. To this end, we prepare a dataset that includes 25-character long sequences in four steps. First, we extract 25-character long non-overlapping sequences from the *E. coli* reference genome (3) shown in Table 1, which we call *sampled sequences* for simplicity. To evenly sample these sequences, each sampled sequence is separated by 75 characters from the previous sampled sequence. Second, our goal is to find *all* sequences in the reference genome that are *similar* and non-identical to the sampled sequences. To achieve this, we use bowtie (4) and find *all* sequences in the *E. coli* reference genome that the sampled sequences align with at least one mismatch and, at most, three mismatches (i.e., at least $\sim 88\%$ similarity). Third, we extract the sequences from the reference regions that the sampled sequences align, which we call *aligned sequences*. Fourth, we prepare our dataset that contains 1,077 FASTA files and 4,130 25-character long sequences overall. Each FASTA file includes 1) a sampled sequence that has at least one alignment in the reference genome based on our mismatching criteria and 2) all aligned sequences that the sampled sequence is aligned to.

To find the non-identical k-mers with the same hash value in each FASTA file, we generate the hash values of all overlapping 16-mers of all sequences in a FASTA file. We use the low-collision hash function that minimap2 uses (i.e., `hash64`) and the `BLEND-I` technique in BLEND to generate these hash values. For BLEND, we use various numbers of neighbors when generating the hash values of 16-mers (see Supplementary Section S1.1 for the relation between the number of neighbors and the seed length, which is 16 in our evaluation). In Supplementary Table S2, we report the number of sequences in our dataset, the number of sequences that have at least one non-identical k-mer pair with the same hash value (i.e., collisions), the ratio of collisions to the overall number of sequences, and the average edit distance between k-mers with collision. We make our dataset available at Zenodo[2], which includes 1,077 FASTA files and the resulting files that we generate the numbers we show in Supplementary Table S2. These resulting files include the non-identical k-mers with the same hash value, the sequence pairs that we extract these k-mers from, and the edit distances with these k-mers.

[1] `https://doi.org/10.5281/zenodo.7317896`
[2] `https://doi.org/10.5281/zenodo.7319786`

Table S2. Fuzzy k-mer matching statistics of sequences that we find using minimap2 and BLEND. The number of overlapping k-mers that BLEND extracts from seed sequences (i.e., neighbors or *n*) are annotated as BLEND-n

Tool	Number of Sequences	Number of Sequences with Collision	Collision/Sequence Ratio	Avg. Edit Distance Between K-mers With Collision
minimap2	4,130	0	0	N/A
BLEND-3	4,130	0	0	N/A
BLEND-5	4,130	11	0.00263663	1.45455
BLEND-7	4,130	50	0.0119847	1.5
BLEND-9	4,130	77	0.0184564	2.01299
BLEND-11	4,130	273	0.0654362	2.80952
BLEND-13	4,130	329	0.0788591	2.20669

S2. A Real Example of Generating the Hash Values of Seeds S_k and S_l

Our goal is to show how the k-mer length k and the number of k-mers to include in a seed, n, affect the final hash value. To this end, we use the following two seeds as found in the Yeast reference genome: S_k : `CGGATGCTACAGTATATACCA` and S_l : `ATGCTACAGTATATACCATCT`. Both seeds are 21-character long. We use two different parameter settings when generating the hash values of these seeds. The first setting uses $k = 7$ as the k-mer length and $n = 15$ as the number of immediately overlapping k-mers to include in a seed so that we can generate the 21-character long seeds S_l and S_k. The second setting uses $k = 15$ as the k-mer length and $n = 7$ as the number of k-mers to include in a seed. We use the `hash64` hash function as provided in the minimap2 implementation to generate the hash values of the k-mers of seeds.

In Supplementary Tables S3 - S10 we show k-mers, the hash values of the k-mers in their binary form, and the gradual change in the counter vectors used to calculate the hash values for seeds S_k and S_l. We update the counter vectors based on the bits in the hash values of each k-mer. Finally, we show the hash values of S_k and S_l in the last rows of each table. In Supplementary Tables S3- S6, we use $k = 7$ as the k-mer length and $n = 15$ as the number of immediately overlapping k-mers to include in a seed. In Supplementary Tables S7- S10, we use $k = 15$ as the k-mer length and $n = 7$ as the number of k-mers to include in a seed.

We make two key observations. First, we observe that the hash values of S_k and S_l are equal ($B(S_k) = B(S_l)$ =`0b11000100 01101100 11101001 10110100`) when we use a short k-mer with high number of neighbors even though these two seeds differ by 3 k-mers. Second, the hash values of these two seeds are not equal when we use fewer neighbors with larger k-mers. For S_k we find the hash value $B(S_k)$ =`0b01101000 01000001 01110100 11000000` and for S_l we find $B(S_l)$ =`0b00101101 10110000 01111100 01010011`. We note that the bit positions with large values in their corresponding counter vectors are less likely to differ between two seeds when the seeds have a large number of k-mers in common. This motivates as to design more intelligent hash functions that are aware of the values in the counter vectors to increase the chance of generating the hash value for similar seeds.

Table S3. Hash Values of the k-mers of seed S_k: `CGGATGCTACAGTATATACCA` for $k = 7$ and $n = 15$. We show the most significant 16 bits of the counter vector $C(S_k)$. Last row shows the most significant 16 bits of the hash value of the seed.

K-mer	Hash Value	C[31]	C[30]	C[29]	C[28]	C[27]	C[26]	C[25]	C[24]	C[23]	C[22]	C[21]	C[20]	C[19]	C[18]	C[17]	C[16]
CGGATGC	0b 10100000 01111111 10000110 10110101	1	-1	1	-1	-1	-1	-1	-1	-1	1	1	1	1	1	1	1
GGATGCT	0b 10101101 11110000 01110100 11010000	2	-2	2	-2	0	0	-2	0	0	2	2	2	0	0	0	0
GATGCTA	0b 01000010 01001011 11011001 10011011	1	-1	1	-3	-1	-1	-1	-1	-1	3	1	1	1	-1	1	1
ATGCTAC	0b 11001100 01110101 01010011 00100110	2	0	0	-4	0	0	-2	-2	-2	4	2	2	0	0	0	2
TGCTACA	0b 10110001 01101010 10101001 10100111	3	-1	1	-3	-1	-1	-3	-1	-3	5	3	1	1	-1	1	1
GCTACAG	0b 11000101 11010111 11010101 00100101	4	0	0	-4	-2	0	-4	0	-2	6	2	2	0	0	2	2
CTACAGT	0b 11001001 01111101 01001010 10110101	5	1	-1	-5	-1	-1	-5	1	-3	7	3	3	1	1	1	3
TACAGTA	0b 00101011 01101111 11111000 11111000	4	0	0	-6	0	-2	-4	2	-4	8	4	2	2	2	2	4
ACAGTAT	0b 11100100 01001110 01110101 00011010	5	1	1	-7	-1	-1	-5	1	-5	9	3	1	3	3	3	3
CAGTATA	0b 10010010 00001101 00100011 10110100	6	0	0	-6	-2	-2	-4	0	-6	8	2	0	4	4	2	4
AGTATAT	0b 01110100 00110000 10101100 00000000	5	1	1	-5	-3	-1	-5	-1	-7	7	3	1	3	3	1	3
GTATATA	0b 11001111 10110000 11001001 10010110	6	2	0	-6	-2	0	-4	0	-6	6	4	2	2	2	0	2
TATATAC	0b 10000001 00001000 00101111 01111111	7	1	-1	-7	-3	-1	-5	1	-7	5	3	1	3	1	-1	1
ATATACC	0b 11001100 11100000 00101000 11011010	8	2	-2	-8	-2	0	-6	0	-6	6	4	0	2	0	-2	0
TATACCA	0b 00110100 00000100 11110100 10010100	7	1	-1	-7	-3	1	-7	-1	-7	5	3	-1	1	1	-3	-1
		B[31]	B[30]	B[29]	B[28]	B[27]	B[26]	B[25]	B[24]	B[23]	B[22]	B[21]	B[20]	B[19]	B[18]	B[17]	B[16]
		1	1	0	0	0	1	0	0	0	1	1	0	1	1	0	0

Best results are highlighted with **bold** text.

Table S4. Hash Values of the k-mers of seed S_k: `CGGATGCTACAGTATATACCA` for $k = 7$ and $n = 15$. We show the least significant 16 bits of the counter vector $C(S_k)$. Last row shows the least significant 16 bits of the hash value of the seed.

K-mer	Hash Value	C[15]	C[14]	C[13]	C[12]	C[11]	C[10]	C[9]	C[8]	C[7]	C[6]	C[5]	C[4]	C[3]	C[2]	C[1]	C[0]
CGGATGC	0b 10100000 01111111 10000110 10110101	1	-1	-1	-1	-1	1	1	-1	1	-1	1	1	-1	1	-1	1
GGATGCT	0b 10101101 11110000 01110100 11010000	0	0	0	0	-2	2	0	-2	2	0	0	2	-2	0	-2	0
GATGCTA	0b 01000010 01001011 11011001 10011011	1	1	-1	1	-1	1	-1	-1	3	-1	-1	3	-1	-1	-1	1
ATGCTAC	0b 11001100 01110101 01010011 00100110	0	2	-2	2	-2	0	0	0	2	-2	0	2	-2	0	0	0
TGCTACA	0b 10110001 01101010 10101001 10100111	1	1	-1	1	-1	-1	-1	1	3	-3	1	1	-3	1	1	1
GCTACAG	0b 11000101 11010111 11010101 00100101	2	2	-2	2	-2	0	-2	2	2	-4	2	0	-4	2	0	2
CTACAGT	0b 11001001 01111101 01001010 10110101	1	3	-3	1	-1	-1	-1	1	3	-5	3	1	-5	3	-1	3
TACAGTA	0b 00101011 01101111 11111000 11111000	2	4	-2	2	0	-2	-2	0	4	-4	4	2	-4	2	-2	2
ACAGTAT	0b 11100100 01001110 01110101 00011010	1	5	-1	3	-1	-1	-3	1	3	-5	3	3	-3	1	-1	1
CAGTATA	0b 10010010 00001101 00100011 10110100	0	4	0	2	-2	-2	-2	2	4	-6	4	4	-4	2	-2	0
AGTATAT	0b 01110100 00110000 10101100 00000000	1	3	1	1	-1	-1	-3	1	3	-7	3	3	-5	1	-3	-1
GTATATA	0b 11001111 10110000 11001001 10010110	2	4	0	0	0	-2	-4	2	4	-8	2	4	-6	2	-2	-2
TATATAC	0b 10000001 00001000 00101111 01111111	1	3	1	-1	1	-1	-3	3	3	-7	3	5	-5	3	-1	-1
ATATACC	0b 11001100 11100000 00101000 11011010	0	2	2	-2	2	-2	-4	2	4	-6	2	6	-4	2	0	-2
TATACCA	0b 00110100 00000100 11110100 10010100	1	3	3	-1	1	-1	-5	1	5	-7	1	7	-5	3	-1	-3
		B[15]	B[14]	B[13]	B[12]	B[11]	B[10]	B[9]	B[8]	B[7]	B[6]	B[5]	B[4]	B[3]	B[2]	B[1]	B[0]
		1	1	1	0	1	0	0	1	1	0	1	1	0	1	0	0

Best results are highlighted with **bold** text.

Table S5. Hash Values of the k-mers of seed S_l: `ATGCTACAGTATATACCATCT` for $k = 7$ and $n = 15$. We show the most significant 16 bits of the counter vector $C(S_l)$. Last row shows the most significant 16 bits of the hash value of the seed.

K-mer	Hash Value	C[31]	C[30]	C[29]	C[28]	C[27]	C[26]	C[25]	C[24]	C[23]	C[22]	C[21]	C[20]	C[19]	C[18]	C[17]	C[16]
ATGCTAC	0b 11001100 01110101 01010011 00100110	1	1	-1	-1	1	1	-1	-1	-1	1	1	1	-1	1	-1	1
TGCTACA	0b 10110001 01101010 10101001 10100111	2	0	0	0	0	0	-2	0	-2	2	2	0	0	0	0	0
GCTACAG	0b 11000101 11010111 11010101 00100101	3	1	-1	-1	-1	1	-3	1	-1	3	1	1	-1	1	1	1
CTACAGT	0b 11001001 01111101 01001010 10110101	4	2	-2	-2	0	0	-4	2	-2	4	2	2	0	2	0	2
TACAGTA	0b 00101011 01101111 11111000 11111000	3	1	-1	-3	1	-1	-3	3	-3	5	3	1	1	3	1	3
ACAGTAT	0b 11100100 01001110 01110101 00011010	4	2	0	-4	0	0	-4	2	-4	6	2	0	2	4	2	2
CAGTATA	0b 10010010 00001101 00100011 10110100	5	1	-1	-3	-1	-1	-3	1	-5	5	1	-1	3	5	1	3
AGTATAT	0b 01110100 00110000 10101100 00000000	4	2	0	-2	-2	0	-4	0	-6	4	2	0	2	4	0	2
GTATATA	0b 11001111 10110000 11001001 10010110	5	3	-1	-3	-1	1	-3	1	-5	3	3	1	1	3	-1	1
TATATAC	0b 10000001 00001000 00101111 01111111	6	2	-2	-4	-2	0	-4	2	-6	2	2	0	2	2	-2	0
ATATACC	0b 11001100 11100000 00101000 11011010	7	3	-3	-5	-1	1	-5	1	-5	3	3	-1	1	1	-3	-1
TATACCA	0b 00110100 00000100 11110100 10010100	6	2	-2	-4	-2	2	-6	0	-6	2	2	-2	0	2	-4	-2
ATACCAT	0b 00000111 10111111 11010101 01001100	5	1	-3	-5	-3	3	-5	1	-5	1	3	-1	1	3	-3	-1
TACCATC	0b 01010110 11100111 00100010 11001101	4	2	-4	-4	-4	4	-4	0	-4	2	4	-2	0	4	-2	0
ACCATCT	0b 00010010 11001000 11001010 11100111	3	1	-5	-3	-5	3	-3	-1	-3	3	3	-3	1	3	-3	-1
		B[31]	B[30]	B[29]	B[28]	B[27]	B[26]	B[25]	B[24]	B[23]	B[22]	B[21]	B[20]	B[19]	B[18]	B[17]	B[16]
		1	1	0	0	0	1	0	0	0	1	1	0	1	1	0	0

Best results are highlighted with **bold** text.

Table S6. Hash Values of the k-mers of seed S_l: `ATGCTACAGTATATACCATCT` for $k = 7$ and $n = 15$. We show the least significant 16 bits of the counter vector $C(S_l)$. Last row shows the least significant 16 bits of the hash value of the seed.

K-mer	Hash Value	C[15]	C[14]	C[13]	C[12]	C[11]	C[10]	C[9]	C[8]	C[7]	C[6]	C[5]	C[4]	C[3]	C[2]	C[1]	C[0]
ATGCTAC	0b 11001100 01110101 01010011 00100110	-1	1	-1	1	-1	-1	1	1	-1	-1	1	-1	-1	1	1	-1
TGCTACA	0b 10110001 01101010 10101001 10100111	0	0	0	0	0	-2	0	2	0	-2	2	-2	-2	2	2	0
GCTACAG	0b 11000101 11010111 11010101 00100101	1	1	-1	1	-1	-1	-1	3	-1	-3	3	-3	-3	3	1	1
CTACAGT	0b 11001001 01111101 01001010 10110101	0	2	-2	0	0	-2	0	2	0	-4	4	-2	-4	4	0	2
TACAGTA	0b 00101011 01101111 11111000 11111000	1	3	-1	1	1	-3	-1	1	1	-3	5	-1	-3	3	-1	1
ACAGTAT	0b 11100100 01001110 01110101 00011010	0	4	0	2	0	-2	-2	2	0	-4	4	0	-2	2	0	0
CAGTATA	0b 10010010 00001101 00100011 10110100	-1	3	1	1	-1	-3	-1	3	1	-5	5	1	-3	3	-1	-1
AGTATAT	0b 01110100 00110000 10101100 00000000	0	2	2	0	0	-2	-2	2	0	-6	4	0	-4	2	-2	-2
GTATATA	0b 11001111 10110000 11001001 10010110	1	3	1	-1	1	-3	-3	3	1	-7	3	1	-5	3	-1	-3
TATATAC	0b 10000001 00001000 00101111 01111111	0	2	2	-2	2	-2	-2	4	0	-6	4	2	-4	4	0	-2
ATATACC	0b 11001100 11100000 00101000 11011010	-1	1	3	-3	3	-3	-3	3	1	-5	3	3	-3	3	1	-3
TATACCA	0b 00110100 00000100 11110100 10010100	0	2	4	-2	2	-2	-4	2	2	-6	2	4	-4	4	0	-4
ATACCAT	0b 00000111 10111111 11010101 01001100	1	3	3	-1	1	-1	-5	3	1	-5	1	3	-3	5	-1	-5
TACCATC	0b 01010110 11100111 00100010 11001101	0	2	4	-2	0	-2	-4	2	2	-4	0	2	-2	6	-2	-4
ACCATCT	0b 00010010 11001000 11001010 11100111	1	3	3	-3	1	-3	-3	1	3	-3	1	1	-3	7	-1	-3
		B[15]	B[14]	B[13]	B[12]	B[11]	B[10]	B[9]	B[8]	B[7]	B[6]	B[5]	B[4]	B[3]	B[2]	B[1]	B[0]
		1	1	1	0	1	0	0	1	1	0	1	1	0	1	0	0

Best results are highlighted with **bold** text.

Table S7. Hash Values of the k-mers of seed S_k: `CGGATGCTACAGTATATACCA` for $k = 15$ and $n = 7$. We show the most significant 16 bits of the counter vector $C(S_k)$. Last row shows the most significant 16 bits of the hash value of the seed.

K-mer	Hash Value	C[31]	C[30]	C[29]	C[28]	C[27]	C[26]	C[25]	C[24]	C[23]	C[22]	C[21]	C[20]	C[19]	C[18]	C[17]	C[16]
CGGATGCTACAGTAT	0b 01001010 11101011 00100110 11001101	-1	1	-1	-1	1	-1	1	-1	1	1	1	-1	1	-1	1	1
GGATGCTACAGTATA	0b 01101100 01000011 11111000 11000000	-2	2	0	-2	2	0	0	-2	0	2	0	-2	0	-2	2	2
GATGCTACAGTATAT	0b 01011000 01000101 00110001 11011000	-3	3	-1	-1	3	-1	-1	-3	-1	3	-1	-3	-1	-1	1	3
ATGCTACAGTATATA	0b 11100001 01110100 01100010 01000010	-2	4	0	-2	2	-2	-2	-2	-2	4	0	-2	-2	0	0	2
TGCTACAGTATATAC	0b 10111100 10011010 00111111 01011011	-1	3	1	-1	3	-1	-3	-3	-1	3	-1	-1	-1	-1	1	1
GCTACAGTATATACC	0b 11101010 01001100 01000100 11100001	0	2	2	-2	4	-2	-2	-4	-2	4	-2	-2	0	0	0	0
CTACAGTATATACCA	0b 00101001 10010001 11111100 01010000	-1	1	3	-3	5	-3	-3	-3	-1	3	-3	-1	-1	-1	-1	1
Seed `CGGATGCTACAGTATATACCA`		B[31]	B[30]	B[29]	B[28]	B[27]	B[26]	B[25]	B[24]	B[23]	B[22]	B[21]	B[20]	B[19]	B[18]	B[17]	B[16]
		0	1	1	0	1	0	0	0	0	1	0	0	0	0	0	1

Best results are highlighted with **bold** text.

Table S8. Hash Values of the k-mers of seed S_k: `CGGATGCTACAGTATATACCA` for $k = 15$ and $n = 7$. We show the least significant 16 bits of the counter vector $C(S_k)$. Last row shows the least significant 16 bits of the hash value of the seed.

K-mer	Hash Value	C[15]	C[14]	C[13]	C[12]	C[11]	C[10]	C[9]	C[8]	C[7]	C[6]	C[5]	C[4]	C[3]	C[2]	C[1]	C[0]
CGGATGCTACAGTAT	0b 01001010 11101011 00100110 11001101	-1	-1	1	-1	-1	1	1	-1	1	1	-1	-1	1	1	-1	1
GGATGCTACAGTATA	0b 01101100 01000011 11111000 11000000	0	0	2	0	0	0	0	-2	2	2	-2	-2	0	0	-2	0
GATGCTACAGTATAT	0b 01011000 01000101 00110001 11011000	-1	-1	3	1	-1	-1	-1	-1	3	3	-3	-1	1	-1	-3	-1
ATGCTACAGTATATA	0b 11100001 01110100 01100010 01000010	-2	0	4	0	-2	-2	0	-2	2	4	-4	-2	0	-2	-2	-2
TGCTACAGTATATAC	0b 10111100 10011010 00111111 01011011	-3	-1	5	1	-1	-1	1	-1	1	5	-5	-1	1	-3	-1	-1
GCTACAGTATATACC	0b 11101010 01001100 01000100 11100001	-4	0	4	0	-2	0	0	-2	2	6	-4	-2	0	-4	-2	0
CTACAGTATATACCA	0b 00101001 10010001 11111100 01010000	-3	1	5	1	-1	1	-1	-3	1	7	-5	-1	-1	-5	-3	-1
Seed `CGGATGCTACAGTATATACCA`		B[15]	B[14]	B[13]	B[12]	B[11]	B[10]	B[9]	B[8]	B[7]	B[6]	B[5]	B[4]	B[3]	B[2]	B[1]	B[0]
		0	1	1	1	0	1	0	0	1	1	0	0	0	0	0	0

Best results are highlighted with **bold** text.

Table S9. Hash Values of the k-mers of seed S_l: `ATGCTACAGTATATACCATCT` for $k = 15$ and $n = 7$. We show the most significant 16 bits of the counter vector $C(S_l)$. Last row shows the most significant 16 bits of the hash value of the seed.

K-mer	Hash Value	C[31]	C[30]	C[29]	C[28]	C[27]	C[26]	C[25]	C[24]	C[23]	C[22]	C[21]	C[20]	C[19]	C[18]	C[17]	C[16]
ATGCTACAGTATATA	0b 11100001 01110100 01100010 01000010	1	1	1	-1	-1	-1	-1	1	-1	1	1	1	-1	1	-1	-1
TGCTACAGTATATAC	0b 10111100 10011010 00111111 01011011	2	0	2	0	0	0	-2	0	0	0	0	2	0	0	0	-2
GCTACAGTATATACC	0b 11101010 01001100 01000100 11100001	3	1	3	-1	1	-1	-1	-1	-1	1	-1	1	1	1	-1	-3
CTACAGTATATACCA	0b 00101001 10010001 11111100 01010000	2	0	4	-2	2	-2	-2	0	0	0	-2	2	0	0	-2	-2
TACAGTATATACCAT	0b 00001110 00100000 11011100 11110110	1	-1	3	-3	3	-1	-1	-1	-1	-1	-1	1	-1	-1	-3	-3
ACAGTATATACCATC	0b 00101111 10111010 00010000 11011111	0	-2	4	-4	4	0	0	0	0	-2	0	2	0	-2	-2	-4
CAGTATATACCATCT	0b 01100101 11100111 10111011 00111011	-1	-1	5	-5	5	1	-1	1	1	-1	1	1	-1	-1	-1	-3
Seed ATGCTACAGTATATACCATCT		B[31]	B[30]	B[29]	B[28]	B[27]	B[26]	B[25]	B[24]	B[23]	B[22]	B[21]	B[20]	B[19]	B[18]	B[17]	B[16]
		0	0	1	0	1	1	0	1	1	0	1	1	0	0	0	0

Best results are highlighted with **bold** text.

Table S10. Hash Values of the k-mers of seed S_l: `ATGCTACAGTATATACCATCT` for $k = 15$ and $n = 7$. We show the least significant 16 bits of the counter vector $C(S_l)$. Last row shows the least significant 16 bits of the hash value of the seed.

K-mer	Hash Value	C[15]	C[14]	C[13]	C[12]	C[11]	C[10]	C[9]	C[8]	C[7]	C[6]	C[5]	C[4]	C[3]	C[2]	C[1]	C[0]
ATGCTACAGTATATA	0b 11100001 01110100 01100010 01000010	-1	1	1	-1	-1	-1	1	-1	-1	1	-1	-1	-1	-1	1	-1
TGCTACAGTATATAC	0b 10111100 10011010 00111111 01011011	-2	0	2	0	0	0	2	0	-2	2	-2	0	0	-2	2	0
GCTACAGTATATACC	0b 11101010 01001100 01000100 11100001	-3	1	1	-1	-1	1	1	-1	-1	3	-1	-1	-1	-3	1	1
CTACAGTATATACCA	0b 00101001 10010001 11111100 01010000	-2	2	2	0	0	2	0	-2	-2	4	-2	0	-2	-4	0	0
TACAGTATATACCAT	0b 00001110 00100000 11011100 11110110	-1	3	1	1	1	3	-1	-3	-1	5	-1	1	-3	-3	1	-1
ACAGTATATACCATC	0b 00101111 10111010 00010000 11011111	-2	2	0	2	0	2	-2	-4	0	6	-2	2	-2	-2	2	0
CAGTATATACCATCT	0b 01100101 11100111 10111011 00111011	-1	1	1	3	1	1	-1	-3	-1	5	-1	3	-1	-3	3	1
Seed ATGCTACAGTATATACCATCT		B[15]	B[14]	B[13]	B[12]	B[11]	B[10]	B[9]	B[8]	B[7]	B[6]	B[5]	B[4]	B[3]	B[2]	B[1]	B[0]
		0	1	1	1	1	1	0	0	0	1	0	1	0	0	1	1

Best results are highlighted with **bold** text.

S3. SIMD Implementation of the SimHash Technique

Supplementary Figure S1 shows the high-level execution flow when calculating the hash value of a seed from its set items that `BLEND-I` or `BLEND-S` identifies, as explained in the *Sequence to Set Conversion* and *Integrating the SimHash Technique* sections. To efficiently perform the bitwise operations in the SimHash technique, BLEND utilizes the SIMD operations in three steps.

First, for each *hash value* in set items, BLEND creates its corresponding *mask* using the `movemask_inverse` function, as shown in ❶. For each bit position t of the hash value, the `movemask_inverse` function assigns the bit at position t of the hash value to the bit position $t*8+7$ of a 256-bit SIMD register (i.e., the most significant bit positions of each 8-bit block), which BLEND uses it as a *mask* in the next steps. We assume 0-based indexing and the mask register is initially 0. Supplementary Figure S2 shows how each bit in hash value propagates to the mask register in ❶. The `movemask_inverse` is an in-house implementation that performs the reverse behavior of the `_mm256_movemask_epi8`[3] SIMD function. Our function efficiently utilizes several other SIMD functions to perform the reverse behavior of `_mm256_movemask_epi8`.

Second, for each mask created in the first step, BLEND updates the values in the counter vector (explained in the *Integrating the SimHash Technique* section), as shown in ❷. To encode the hash value into its vector representation, BLEND uses the `_mm256_blendv_epi8`[4] SIMD function with 1) the mask register BLEND creates in the first step, 2) two 256-bit wide SIMD registers that include 32× 8-bit integers. For the first register, all 8-bit values are initialized to 1, and for the second register, all 8-bit values are initialized to -1. The `_mm256_blendv_epi8` function generates a new 256-bit register with 8-bit integers where each 8-bit block is copied from either the first or the second register based on the most significant value in the mask register. If the most significant value in the mask register is 0, the corresponding 8-bit block in the first register is copied. Otherwise, the 8-bit block in the second register is copied. We show in detail how the values in these registers propagate to the resulting 256-bit register in Supplementary Figure S2 in ❷. BLEND, then, performs addition using the `_mm256_adds_epi8`[5] function between the register that the `_mm256_blendv_epi8` function generates and the 256-bit *counter vector* that includes 32× 8-bit integers. We assume that all the 8-bit values in the counter vector are initially 0. BLEND keeps updating the counter vector as it iterates through the set items (i.e., hash values). The resulting value is written back to the counter vector to use it in the next iterations with the next set item. We note that the current design encodes 1 bits as -1 and 0 bits as 1, which is the opposite case of our explanation in the *Integrating the SimHash Technique* section. Although we perform our encoding in an opposite way, BLEND generates the final hash values as originally explained. The reason for such a design change is due to the behavior of the function we use in the third step.

Third, BLEND converts the final result in the counter vector to its corresponding 32-bit hash value of a set (explained in the decoding step of the *Integrating the SimHash Technique* section), as shown in ❸. BLEND uses the `_mm256_movemask_epi8` function that takes a 256-bit register of 8-bit blocks and assigns the corresponding bit accordingly in a 32-bit value. The behavior of this function is essentially the reverse behavior of the `movemask_inverse` function that we explain in the first step (i.e., reversing the arrows in Supplementary Figure S2 ❶ can simulate the `_mm256_movemask_epi8` function). Thus, it assigns 1 to the bit position t of a hash value if the bit at position $t*8+7$ (i.e., the most significant bit of an 8-bit integer) is 1. Since the most significant is 1 *only* for the negative values according to the signed integer value convention, this function creates the opposite behavior of our decoding step we explain in the *Integrating the SimHash Technique* section. We resolve this issue by performing the encoding in an opposite way in the second step, where the resulting counter vector includes negative values when the majority of bits at a bit position is 1. Thus, the final hash value contains the same bits as explained in our main paper.

Although we omit the details here, BLEND avoids performing redundant computations when calculating the hash values of each input sequence, as the set items between each of these input sequences are likely to be shared. For example, minimap2 generates a hash value for each k-mer in a sequence and selects the k-mer with the minimum hash value in a window of k-mers as the minimizer, as shown in Figure 2. Assuming that the set items of each k-mer are l-mers, each k-mer differs by two l-mers at most with its next overlapping k-mer: one different l-mer contains the leading character of the first k-mer, and the other contains the trailing character of the next k-mer. Since there are two l-mer changes at most, BLEND calculates only the difference between subsequent k-mers. Thus, BLEND keeps a buffer in a first-in, first-out fashion such that the corresponding encoded hash value of the l-mer that is missing in the next k-mer is subtracted from the counter vector and popped from the queue while performing an addition *only* for the l-mer that is missing from the previous k-mer and pushing it into the queue.

We perform our operations on 256-bit wide SIMD registers. BLEND works on 8-bit integer blocks assigned for each bit in a hash value. Since our registers are 256-bit wide, BLEND uses 32-bit hash values when calculating the SimHash value of a seed. Our implementation allows working on up to 64-bit hash values by dividing the most and least significant 32 bits into two 32-bit hash values. Each 32-bit hash value can independently follow the three steps we show in Supplementary Figure S1, and the final 64-bit value can be generated by the shift operations between the final 32-bit hash values. Although our approach is scalable to allow hash values with a larger number of bits, the current implementation does not support such flexible scaling and works on up to 64-bits.

---

[3] https://software.intel.com/sites/landingpage/IntrinsicsGuide/#text=_mm256_movemask_epi8&ig_expand=4874

[4] https://software.intel.com/sites/landingpage/IntrinsicsGuide/#text=_mm256_blendv_epi8&ig_expand=515

[5] https://software.intel.com/sites/landingpage/IntrinsicsGuide/#text=_mm256_adds_epi8&ig_expand=220

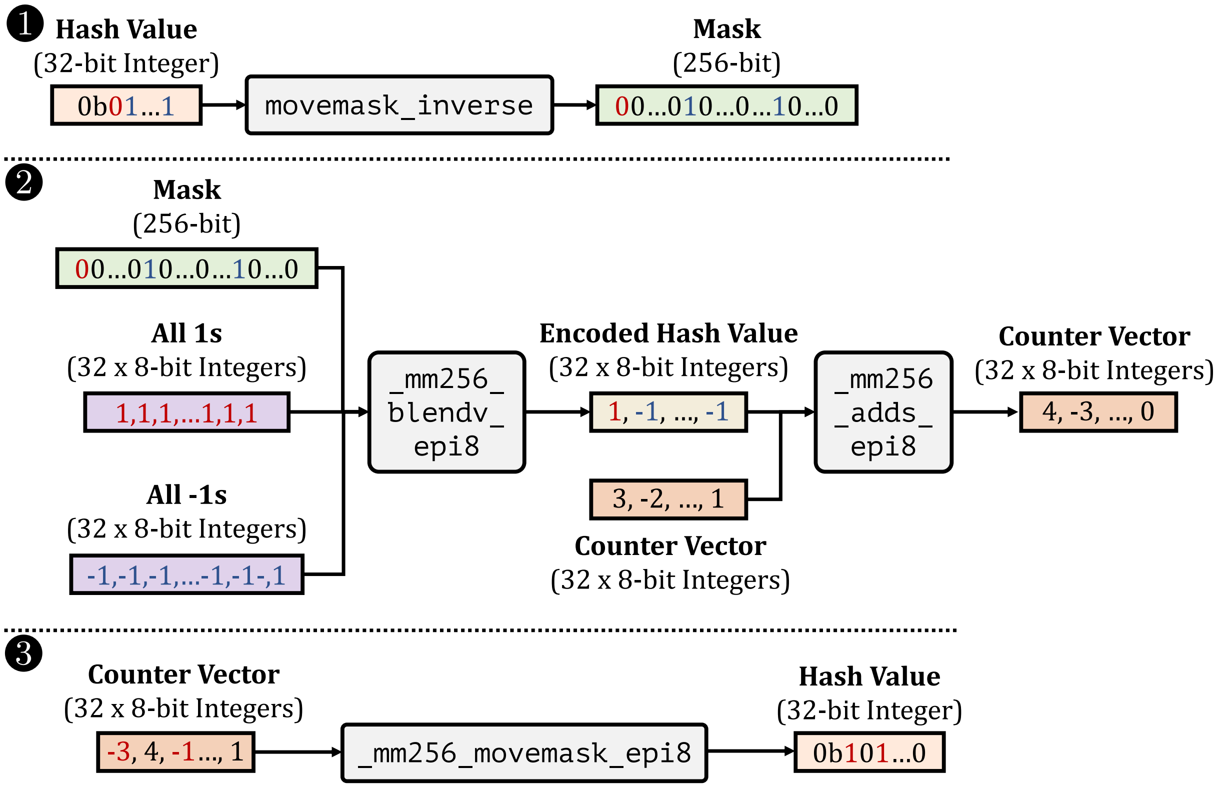

Figure S1. SIMD execution flow when generating the hash value of a seed from its set items that `BLEND-I` or `BLEND-S` identifies. Colors highlight the propagation of bits and values to the outputs of functions.

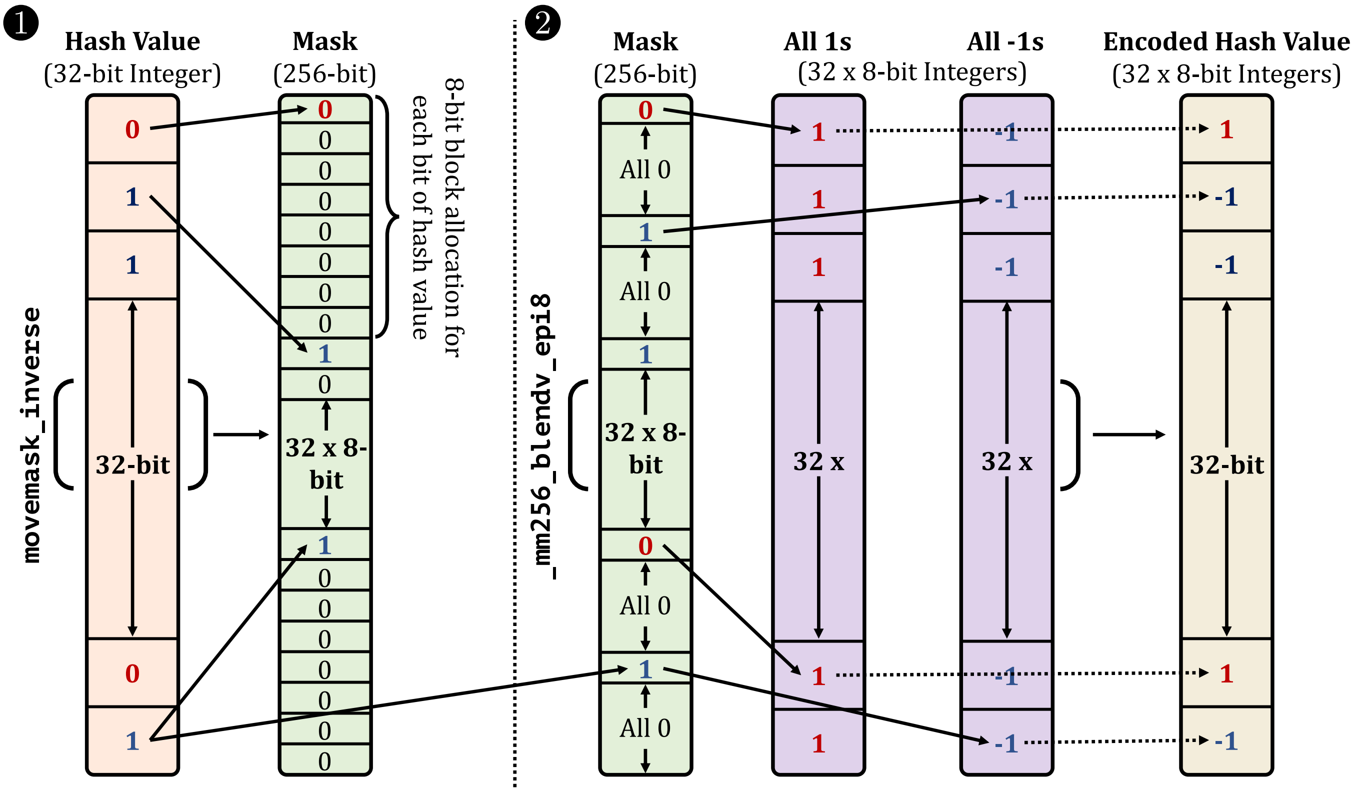

Figure S2. Details of the `movemask_inverse` and `_mm256_blendv_epi8` executions. Colors and arrows highlight the propagation of bits and values to the outputs of functions.

S4. Parameter Exploration

S4.1. The trade-off between BLEND-I and BLEND-S

Our goal is to show the performance and accuracy trade-offs between the seeding techniques that BLEND supports: BLEND-I and BLEND-S. In Supplementary Figures S3 and S4, we show the performance and peak memory usage comparisons when using BLEND-I and BLEND-S as the seeding technique by keeping all the other relevant parameters identical (e.g., number of k-mers to include in a seed *n*, window length *w*). In Supplementary Table S11, we show the assembly quality comparisons in terms of the accuracy and contiguity of the assemblies that we generate using the overlaps that BLEND-I and BLEND-S find. In Supplementary Tables S12 and S13, we show the read mapping quality and accuracy results using these two seeding techniques, respectively.

We also show the values for different parameters we test with BLEND in Supplementary Table S14. We determine the default parameters of BLEND empirically based on the combination of best performance, memory overhead, and accuracy results.

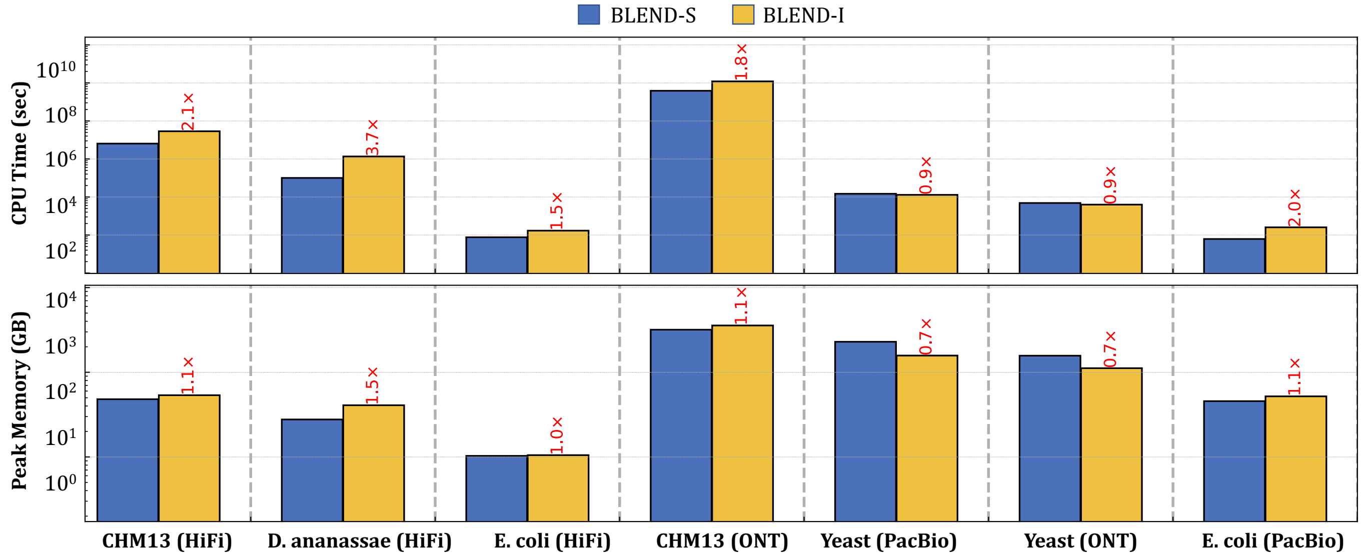

Figure S3. CPU time and peak memory footprint comparisons of read overlapping.

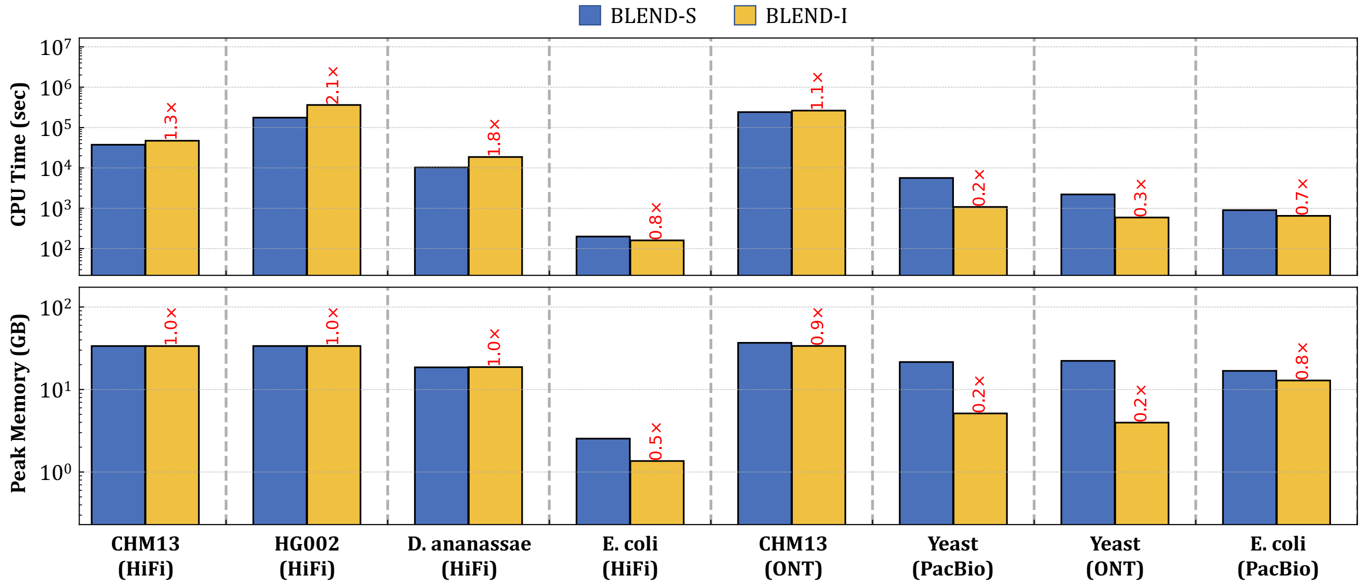

Figure S4. CPU time and peak memory footprint comparisons of read mapping.

Table S11. Assembly quality comparisons between `BLEND-I` and `BLEND-S`.

Dataset	Tool	Average Identity (%)	Genome Fraction (%)	K-mer Compl. (%)	Aligned Length (Mbp)	Mismatch per 100Kbp (#)	Average GC (%)	Assembly Length (Mbp)	Largest Contig (Mbp)	NGA50 (Kbp)	NG50 (Kbp)
CHM13 (HiFi)	`BLEND-I`	99.7535	96.7203	83.65	3,054.49	48.49	**40.79**	**3,059.29**	**41.8342**	**8,507.53**	**8,508.92**
	`BLEND-S`	**99.8526**	**98.4847**	**90.15**	**3,092.54**	**22.02**	40.78	3,095.21	22.8397	5,442.25	5,442.31
	Reference	100	100	100	3,054.83	0.00	40.85	3,054.83	248.387	154,260	154,260
D. ananassae (HiFi)	`BLEND-I`	99.6890	97.2290	77.85	**270.218**	233.18	41.95	280.388	5.01099	356.745	356.745
	`BLEND-S`	**99.7856**	**97.2308**	**86.43**	240.391	**143.13**	**41.75**	**247.153**	**6.23256**	**792.407**	**798.913**
	Reference	100	100	100	213.805	0.00	41.81	213.818	30.6728	26,427.4	26,427.4
E. coli (HiFi)	`BLEND-I`	99.6902	**99.8824**	79.36	5.04157	17.92	**50.52**	**5.04263**	**4.94601**	**4,025.48**	**4,946.01**
	`BLEND-S`	**99.8320**	99.8801	**87.91**	**5.12155**	**3.77**	50.53	5.12155	3.41699	3,416.99	3,416.99
	Reference	100	100	100	5.04628	0.00	50.52	5.04628	4.94446	4,944.46	4,944.46
CHM13 (ONT)	`BLEND-I`	N/A	N/A	**29.26**	**2,891.28**	4,077.53	**41.32**	**2,897.87**	**25.2071**	**5,061.52**	**5,178.59**
	`BLEND-S`	N/A	N/A	0	0.010546	**3,250.70**	51.30	0.010548	0.010548	0	0
	Reference	100	100	100	3,117.29	0.00	40.75	3,117.29	248.387	150,617	150,617
Yeast (PacBio)	`BLEND-I`	89.1677	**97.0854**	**33.81**	12.3938	**2,672.37**	38.84	**12.4176**	**1.54807**	**635.966**	**636.669**
	`BLEND-S`	**90.3347**	83.8814	33.17	**22.9473**	4,795.58	**38.71**	22.9523	0.265118	114.125	116.143
	Reference	100	100	100	12.1571	0.00	38.15	12.1571	1.53193	924.431	924.431
Yeast (ONT)	`BLEND-I`	89.6889	**99.2974**	**35.95**	**12.3222**	2,529.47	38.64	**12.3225**	**1.10582**	**793.046**	**793.046**
	`BLEND-S`	**91.0865**	7.9798	4.90	0.898565	**2,006.91**	**38.35**	0.899654	0.043321	0	0
	Reference	100	100	100	12.1571	0.00	38.15	12.1571	1.53193	924.431	924.431
E. coli (PacBio)	`BLEND-I`	88.5806	**96.5238**	**32.32**	**5.90024**	1,857.56	**49.81**	**6.21598**	**2.40671**	**769.981**	**2,060.4**
	`BLEND-S`	**90.3551**	36.6230	17.07	2.10137	**1,299.50**	48.91	2.10704	0.095505	0	0
	Reference	100	100	100	5.6394	0.00	50.43	5.6394	5.54732	5,547.32	5,547.32

Best results are highlighted with **bold** text. For most metrics, the best results are the ones closest to the corresponding value of the reference genome.
The best results for *Aligned Length* are determined by the highest number within each dataset. We do not highlight the reference results as the best results.
N/A indicates that we could not generate the corresponding result because tool, QUAST, or dnadiff failed to generate the statistic.

Table S12. Read mapping quality comparisons between `BLEND-I` and `BLEND-S`.

Dataset	Tool	Average Depth of Cov. (×)	Breadth of Coverage (%)	Aligned Reads (#)	Properly Paired (%)
CHM13 (HiFi)	`BLEND-I`	16.58	99.991	**3,172,305**	NA
	`BLEND-S`	16.58	99.991	3,171,916	NA
HG002 (HiFi)	`BLEND-I`	**51.25**	**92.245**	6,813,886	NA
	`BLEND-S`	11.24	13.860	**11,424,762**	NA
D. ananassae (HiFi)	`BLEND-I`	**57.51**	99.650	**1,249,666**	NA
	`BLEND-S`	57.37	**99.662**	1,223,388	NA
E. coli (HiFi)	`BLEND-I`	99.14	99.897	**39,064**	NA
	`BLEND-S`	99.14	99.897	39,048	NA
CHM13 (ONT)	`BLEND-I`	**29.34**	**99.999**	**10,322,767**	NA
	`BLEND-S`	17.51	99.700	5,760,401	NA
Yeast (PacBio)	`BLEND-I`	**195.87**	**99.980**	**270,064**	NA
	`BLEND-S`	142.31	99.975	179,039	NA
Yeast (ONT)	`BLEND-I`	**97.88**	**99.964**	**134,919**	NA
	`BLEND-S`	59.57	99.906	75,110	NA
E. coli (PacBio)	`BLEND-I`	**97.51**	100	**83,924**	NA
	`BLEND-S`	56.87	100	40,694	NA

Best results are highlighted with **bold** text.
Properly paired rate is only available for paired-end Illumina reads.

Table S13. Read mapping accuracy comparisons between `BLEND-I` and `BLEND-S`.

Dataset	Overall Error Rate (%)	
	`BLEND-I`	`BLEND-S`
CHM13 (ONT)	**1.5168427**	5.996888
Yeast (PacBio)	**0.2403134**	0.6959378
Yeast (ONT)	**0.2386617**	0.6284117

Best results are highlighted with **bold** text.

Table S14. Performance, memory, and accuracy comparisons using different parameter settings in BLEND.

Tool	K-mer Length (k)	# of k-mers in a Seed (n)	Window Length (w)	CPU Time (seconds)	Peak Memory (KB)	Average Identity (%)	Genome Fraction (%)
BLEND	9	11	200	62.38	1,115,384	99.7255	99.8502
BLEND	9	13	200	58.13	994,120	99.7294	99.7808
BLEND	9	15	200	49.79	1,030,148	99.7411	99.7619
BLEND	9	17	200	45.03	960,080	99.7302	99.7460
BLEND	9	21	200	36.84	976,456	99.7257	99.6640
BLEND	15	5	200	83.05	1,168,612	99.6735	99.7625
BLEND	15	7	200	74.93	1,137,360	99.7009	99.5874
BLEND	15	11	200	58.09	1,051,912	99.7149	99.1166
BLEND	19	5	200	77.16	1,130,604	99.7312	99.8802
BLEND	19	7	200	50.50	1,078,596	99.7880	99.8424
BLEND	19	11	200	46.26	977,060	99.8078	99.6438
BLEND	21	5	200	67.85	1,116,684	99.7472	99.8835
BLEND	21	7	200	61.63	1,042,724	99.7969	99.8605
BLEND	21	11	200	42.35	969,184	99.8340	99.7515
BLEND	25	5	200	65.61	1,057,804	99.7769	99.8818
BLEND	25	7	200	54.88	1,029,888	99.8320	99.8801
BLEND	25	11	200	37.01	936,260	99.8646	99.8001
BLEND	25	15	200	29.83	866,208	99.8838	99.7307
BLEND	25	17	200	29.59	826,456	99.8784	99.7521
BLEND	25	21	200	26.09	791,736	99.8774	99.6955
BLEND	9	11	50	263.82	1,786,516	99.7013	99.8612
BLEND	9	13	50	411.24	1,805,800	99.6995	99.8573
BLEND	9	15	50	271.00	1,729,784	99.6798	99.8517
BLEND	9	17	50	238.52	1,690,912	99.6690	99.8083
BLEND	9	21	50	206.76	1,725,168	99.6496	99.8150
BLEND	15	5	50	330.84	1,785,456	99.6634	99.8604
BLEND	15	7	50	337.95	1,812,052	99.6280	99.8177
BLEND	15	11	50	236.82	1,803,816	99.5831	99.6893
BLEND	19	5	50	328.67	1,692,248	99.7077	99.8794
BLEND	19	7	50	295.57	1,713,940	99.7188	99.8579
BLEND	19	11	50	201.79	1,700,412	99.7015	99.8578
BLEND	21	5	50	378.58	1,625,388	99.7120	99.8832
BLEND	21	7	50	278.56	1,695,476	99.7333	99.8832
BLEND	21	11	50	189.33	1,694,820	99.7623	99.8594
BLEND	25	5	50	323.69	1,685,304	99.7272	99.8831
BLEND	25	7	50	211.78	1,647,984	99.7722	99.8831
BLEND	25	11	50	170.60	1,683,736	99.8094	99.8866
BLEND	25	15	50	142.42	1,622,452	99.8170	99.8576
BLEND	25	17	50	103.96	1,590,776	99.8073	99.8206
BLEND	25	21	50	109.62	1,548,228	99.7792	99.7880
BLEND	9	11	20	837.50	2,769,552	99.6916	99.8784
BLEND	9	13	20	813.50	2,765,480	99.6834	99.8785
BLEND	9	15	20	764.91	2,795,848	99.6797	99.8756
BLEND	9	17	20	739.52	2,823,188	99.6801	99.8802

We use the *E.coli* dataset for all these runs

S4.2. The trade-off between BLEND and minimap2

Our goal is to compare BLEND and minimap2 using the same set of parameters that BLEND uses when generating its results. To achieve this, we control the following two conditions. First, we ensure that we use the same seeding technique that minimap2 uses. To this end, we use the `BLEND-I` seeding technique, which uses minimizers as seeds. We should note that `BLEND-I` does not always provide the best results in terms of performance or accuracy for the HiFi reads as the default seeding technique is `BLEND-S` for HiFi datasets in BLEND.

Second, we use the same seed length when we compare BLEND with minimap2. In minimap2, the seed length is the same as the k-mer length as minimap2 finds the minimizer k-mers from the hash values of k-mers. The seed length in `BLEND-I` is determined by *both* the k-mer length and the number of k-mers that we include in a seed (i.e., n). For example, BLEND uses the `BLEND-I` seeding technique with the k-mer length $k = 19$ and the number of neighbors $n = 5$ for the PacBio reads. Combining immediately overlapping 5-many 19-mers generates seeds with length $19 + 5 - 1 = 23$. Thus, `BLEND-I` uses seeds of length 23 based on these parameters. Supplementary Table S16 shows the seed length calculation for both `BLEND-I` and `BLEND-S`. We calculate the seed lengths for the datasets where BLEND uses `BLEND-I` as the default option (i.e., the PacBio and ONT datasets) in read overlapping. We note that BLEND uses the same seed length and window length as in minimap2 for mapping long reads. Thus, we do not report the read mapping results in this section, which are already reported in the main paper when comparing BLEND with minimap2. To run minimap2 with the same parameter conditions, we apply the same seed length and the window length that BLEND uses to minimap2 using the k and w parameters, respectively. We show these parameters in Supplementary Table S17 (minimap-Eq). In the results we show below, minimap-Eq indicates the runs of minimap2 when using the same set of parameters that BLEND uses with the `BLEND-I` technique.

In Supplementary Figure S5, we show the performance and peak memory usage comparisons when using BLEND with the `BLEND-I` seeding technique, minimap2, and minimap2-Eq. In Supplementary Table S15, we show the assembly quality comparisons in terms of the accuracy and contiguity of the assemblies that we generate using the overlaps that each tool finds.

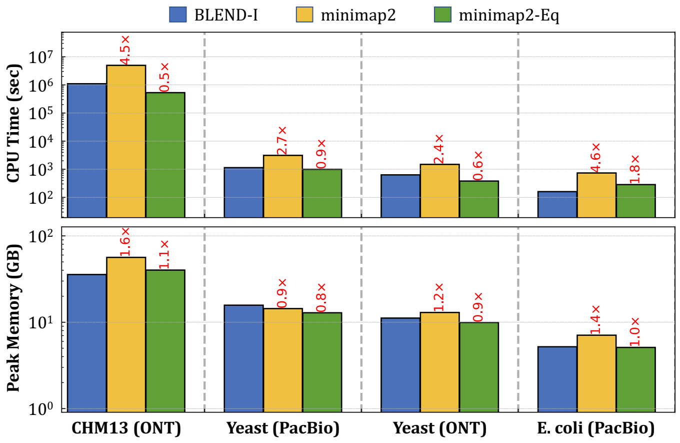

Figure S5. CPU time and peak memory footprint comparisons of read overlapping.

Table S15. Assembly quality comparisons when using the parameters equivalent to `BLEND-I`.

Dataset	Tool	Average Identity (%)	Genome Fraction (%)	K-mer Compl. (%)	Aligned Length (Mbp)	Mismatch per 100Kbp (#)	Average GC (%)	Assembly Length (Mbp)	Largest Contig (Mbp)	NGA50 (Kbp)	NG50 (Kbp)
CHM13 (ONT)	`BLEND-I`	N/A	N/A	29.26	2,891.28	4,077.53	**41.32**	2,897.87	25.2071	5,061.52	5,178.59
	minimap2	N/A	N/A	28.32	2,860.26	4,660.73	41.36	**2,908.55**	**66.7564**	**13,189.2**	**13,820.3**
	minimap2-Eq	N/A	N/A	**29.32**	**3,117.29**	**4,025.22**	**41.32**	2,882.94	24.6651	3,634.05	3,653.47
	Reference	100	100	100	3,117.29	0.00	40.75	3,117.29	248.387	150,617	150,617
Yeast (PacBio)	`BLEND-I`	89.1677	97.0854	33.81	**12.3938**	2,672.37	38.84	12.4176	1.54807	635.966	636.669
	minimap2	88.9002	96.9709	33.38	12.0128	2,684.38	38.85	**12.3325**	**1.56078**	**810.046**	**828.212**
	minimap2-Eq	**89.2166**	**97.2674**	**33.93**	12.3886	**2,653.08**	**38.82**	12.4241	1.53435	643.136	781.136
	Reference	100	100	100	12.1571	0.00	38.15	12.1571	1.53193	924.431	924.431
Yeast (ONT)	`BLEND-I`	**89.6889**	99.2974	**35.95**	**12.3222**	2,529.47	**38.64**	**12.3225**	1.10582	793.046	793.046
	minimap2	88.9393	**99.6878**	34.84	12.304	2,782.59	38.74	12.3725	**1.56005**	**796.718**	**941.588**
	minimap2-Eq	89.6653	97.3273	35.62	11.826	**2,465.87**	**38.64**	11.8282	1.07367	605.201	677.415
	Reference	100	100	100	12.1571	0.00	38.15	12.1571	1.53193	924.431	924.431
E. coli (PacBio)	`BLEND-I`	88.5806	96.5238	32.32	**5.90024**	1,857.56	**49.81**	6.21598	2.40671	769.981	2,060.4
	minimap2	88.1365	92.7603	30.74	5.37728	2,005.72	49.66	**6.02707**	3.77098	367.442	3,770.98
	minimap2-Eq	**88.6371**	**96.8540**	**32.33**	5.82218	**1,816.29**	49.76	6.05821	**3.77318**	**1,119.04**	**3,773.18**
	Reference	100	100	100	5.6394	0.00	50.43	5.6394	5.54732	5,547.32	5,547.32

Best results are highlighted with **bold** text. For most metrics, the best results are the ones closest to the corresponding value of the reference genome.
The best results for *Aligned Length* are determined by the highest number within each dataset. We do not highlight the reference results as the best results.
N/A indicates that we could not generate the corresponding result because tool, QUAST, or dnadiff failed to generate the statistic.

S5. The Genome-wide Coverage Comparison

We map the HG002 reads to the human reference genome (GRCh37) using BLEND and minimap2. Supplementary Figures S6 and S7 show the depth of mapping coverage at each position of the reference genome chromosomes for BLEND and minimap2 on the left and right sides of the figures, respectively. To calculate the position-wise depth of coverage, we use the `multiBamSummary` tool from the deepTools2 package (5). The `multiBamSummary` tool divides the reference genome into consecutive bins of equal size (10,000 bases) to calculate the genome-wide coverage in fine granularity. For positions where the coverage is higher than 500×, we set the coverage to 500× for visibility reasons as there are only a negligible amount of such regions where either BLEND or minimap2 exceeds this threshold without the other one exceeding it.

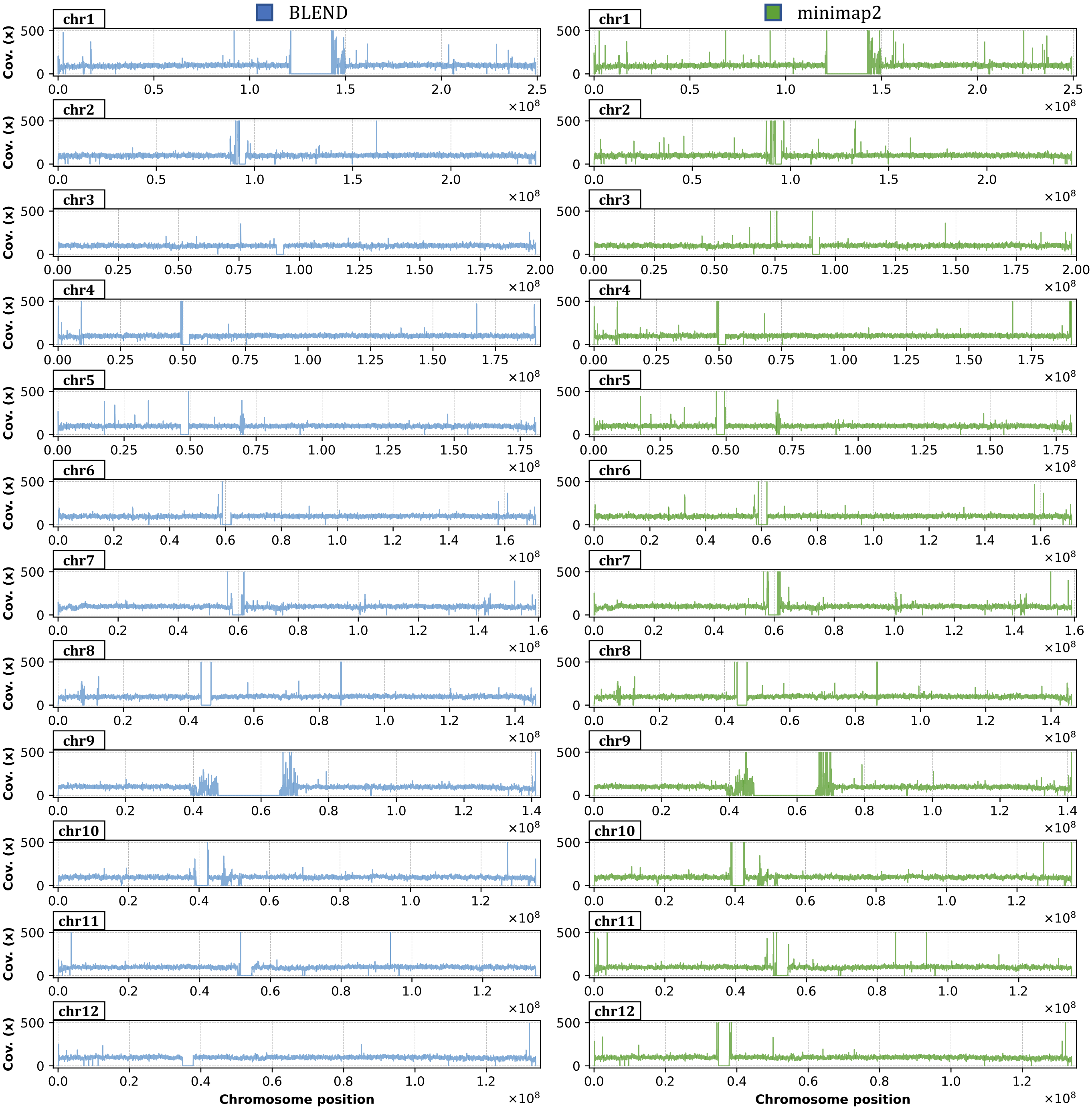

Figure S6. Depth of coverage at each position (binned) of the GRCh37 reference genome (chromosomes 1 to 12) after mapping the HG002 reads using BLEND and minimap2. We label the chromosomes on the top left corner of each plot.

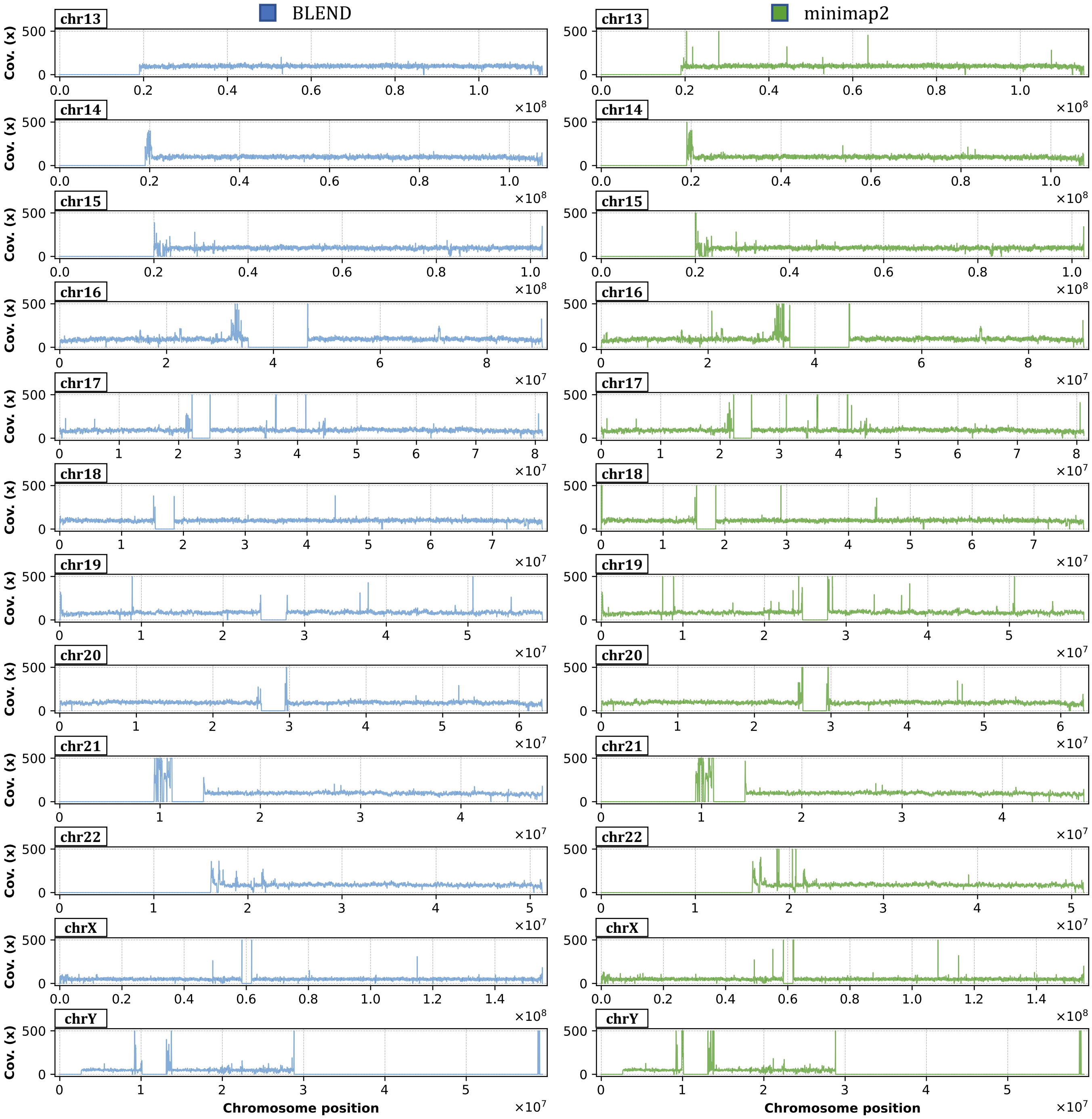

Figure S7. Depth of coverage at each position (binned) of the GRCh37 reference genome (chromosomes 13 to Y) after mapping the HG002 reads using BLEND and minimap2. We label the chromosomes on the top left corner of each plot.

To find the positions where the depth of coverage significantly differs between BLEND and minimap2, we subtract the minimap2 coverage from the BLEND coverage for each chromosome position that we show in Figures S6 and S7. We show the coverage differences in Figure S8, where the positive values show the positions that minimap2 has a higher depth of coverage than BLEND, and negative values show the positions that BLEND has a higher coverage.

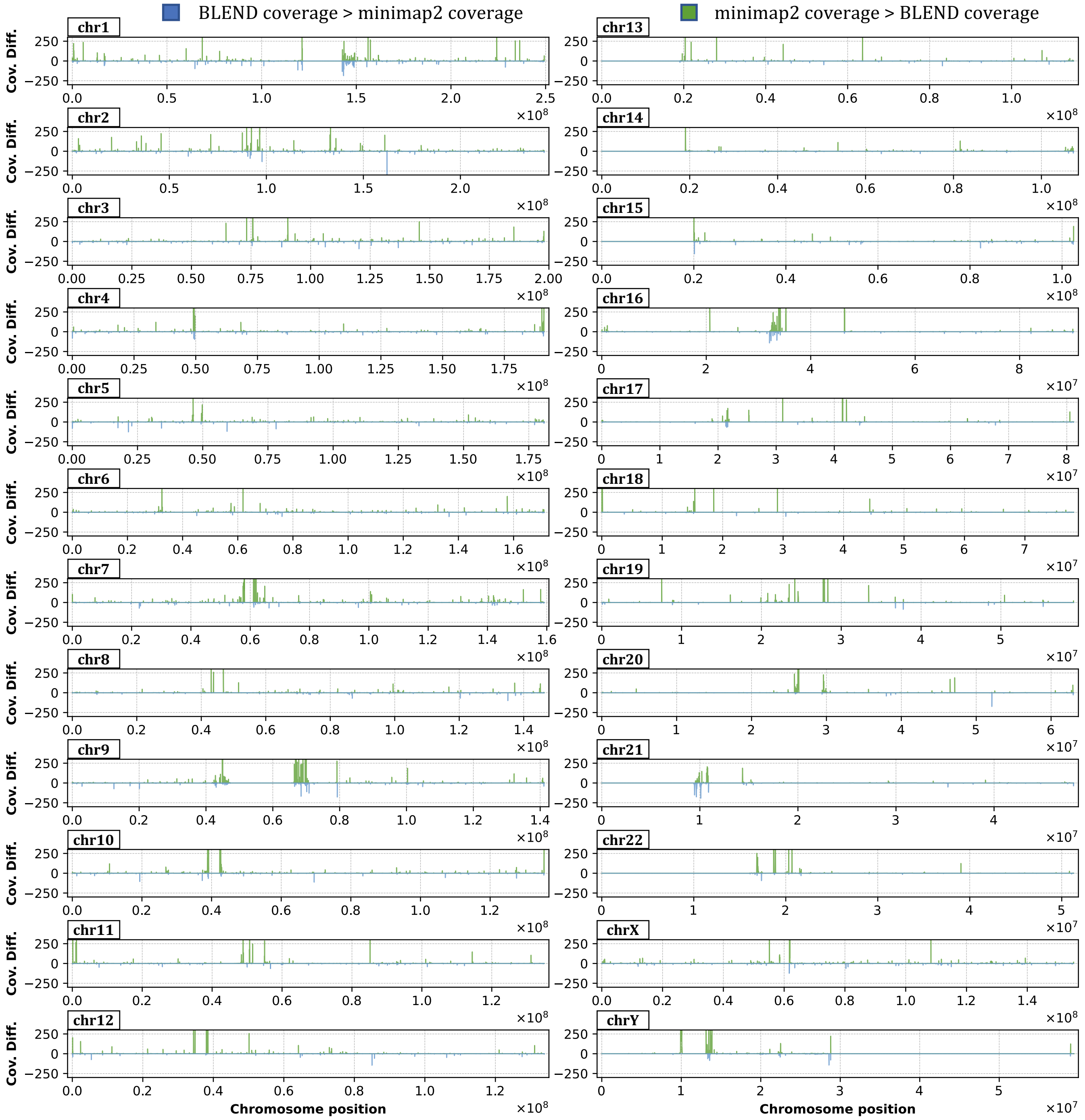

Figure S8. Difference between the depth of coverage of minimap2 and BLEND. Positive values show the positions where minimap2 has higher coverage and negative values show the positions where BLEND has higher coverage. We label the chromosomes on the top left corner of each plot.

S6. Parameters and Tool Versions

Supplementary Table S16 shows the parameters we use in BLEND and their definition. Since BLEND uses the minimap2 implementation as a baseline, the rest of the parameters we do not show in Supplementary Table S16 can be found on the manual page of minimap2[6]. In Supplementary Table S17, we show the parameters we use with BLEND, minimap2, and MHAP (6) for read overlapping. Since there are no default parameters for minimap2 and MHAP when using the HiFi reads, we used the parameters as suggested by the HiCanu tool (7). We found these parameters in the source code of Canu. For minimap2 and MHAP, the HiFi parameters are found in the GitHub pages[7,8], respectively. In Supplementary Table S17, *minimap-Eq* shows the parameters that are equivalent to the parameters we use with BLEND without the fuzzy seed matching capability.

In Supplementary Table S18, we show the parameters we use with BLEND, minimap2 (8), LRA (9), Winnowmap2 (10, 11), S-conLSH (12, 13), and Strobealign (14) for read mapping.

In Supplementary Table S19, we show the version numbers of each tool. When calculating the performance and peak memory usage, we use the time command from Linux and append the following command to the beginning of each of our runs: `/usr/bin/time -vp`.

Table S16. Definition of parameters in BLEND

Parameter	Definition
–strobemers	Use the `BLEND-S` mechanism when generating the list of k-mers of a seed
–immediate	Use the `BLEND-I` mechanism when generating the list of k-mers of a seed
-H	Use homopolymer-compressed k-mers
-w INT	Window size used when finding minimizers.
-k INT	k-mer size used when generating the list of k-mers of a seed
–neighbors INT	Number of k-mers included in the list of seeds.<br>Combination of both -k (k) and –neighbors (n) determines the seed length.<br>Seed length in `BLEND-S` is calculated as: $k \times n$<br>Seed length in `BLEND-I` is calculated as: $k+(n-1)$
–fixed-bits INT	Bit length of hash values that BLEND generates for each seed.<br>Setting it to $2 \times k$ is the default behavior.
-t INT	Number of CPU threads to use.
-x STR	Preset for setting the default parameters given the use case (STR)
-x map-ont	Preset for mapping ONT reads. It uses the following parameters:<br>–immediate -w 10 -k 9 –neighbors 7 –fixed-bits 30
-x map-pb	Preset for mapping erroneous PacBio reads. It uses the following parameters:<br>–immediate -H -w 10 -k 13 –neighbors 7 –fixed-bits 32
-x map-hifi	Preset for mapping accurate long (HiFi) reads. It uses the following parameters:<br>–strobemers -w 50 -k 19 –neighbors 5 –fixed-bits 38
-x sr	Preset for mapping short reads. It uses the following parameters:<br>–immediate -w 11 -k 21 –neighbors 5 –fixed-bits 32
-x ava-ont	Preset for overlapping ONT reads. It uses the following parameters:<br>–immediate -w 10 -k 15 –neighbors 5 –fixed-bits 30
-x ava-pb	Preset for overlapping erroneous PacBio reads. It uses the following parameters:<br>–immediate -H -w 10 -k 19 –neighbors 5 –fixed-bits 38
-x ava-hifi	Preset for overlapping accurate long (HiFi) reads. It uses the following parameters:<br>–strobemers -w 200 -k 25 –neighbors 7 –fixed-bits 50

[6] `https://lh3.github.io/minimap2/minimap2.html`

[7] `https://github.com/marbl/canu/blob/404540a944664cfab00617f4f4fa37be451b34e0/src/pipelines/canu/OverlapMMap.pm#L63-L65`

[8] `https://github.com/marbl/canu/blob/404540a944664cfab00617f4f4fa37be451b34e0/src/pipelines/canu/OverlapMhap.pm#L100-L131`

Table S17. Parameters* we use in our evaluation for each tool and dataset in read overlapping.

Tool	Dataset	Parameters
BLEND	*CHM13 (HiFi)*	-x ava-hifi -t 32
BLEND	*D. ananassae (HiFi)*	-x ava-hifi -t 32
BLEND	*E. coli (HiFi)*	-x ava-hifi -t 32
BLEND	*CHM13 (ONT)*	-x ava-ont -t 32
BLEND	*Yeast (PacBio)*	-x ava-pb -t 32
BLEND	*Yeast (ONT)*	-x ava-ont -t 32
BLEND	*E. coli (PacBio)*	-x ava-pb -t 32
minimap2	*CHM13 (HiFi)*	-x ava-pb -Hk21 -w14 -t 32
minimap2	*D. ananassae (HiFi)*	-x ava-pb -Hk21 -w14 -t 32
minimap2	*E. coli (HiFi)*	-x ava-pb -Hk21 -w14 -t 32
minimap2	*CHM13 (ONT)*	-x ava-ont -t 32
minimap2	*Yeast (PacBio)*	-x ava-pb -t 32
minimap2	*Yeast (ONT)*	-x ava-ont -t 32
minimap2	*E. coli (PacBio)*	-x ava-pb -t 32
minimap2-Eq	*CHM13 (ONT)*	-x ava-ont -k19 -w10 -t 32
minimap2-Eq	*Yeast (PacBio)*	-x ava-pb -k23 -w10 -t 32
minimap2-Eq	*Yeast (ONT)*	-x ava-ont -k19 -w10 -t 32
minimap2-Eq	*E. coli (PacBio)*	-x ava-pb -k23 -w10 -t 32
MHAP	*CHM13 (HiFi)*	–store-full-id –ordered-kmer-size 18 –num-hashes 128 –num-min-matches 5 –ordered-sketch-size 1000 –threshold 0.95 –num-threads 32
MHAP	*D. ananassae (HiFi)*	–store-full-id –ordered-kmer-size 18 –num-hashes 128 –num-min-matches 5 –ordered-sketch-size 1000 –threshold 0.95 –num-threads 32
MHAP	*E. coli (HiFi)*	–store-full-id –ordered-kmer-size 18 –num-hashes 128 –num-min-matches 5 –ordered-sketch-size 1000 –threshold 0.95 –num-threads 32
MHAP	*Yeast (PacBio)*	–store-full-id –num-threads 32
MHAP	*Yeast (ONT)*	–store-full-id –num-threads 32
MHAP	*E. coli (PacBio)*	–store-full-id –num-threads 32

* For the definitions of the parameters we use in BLEND, please see Supplementary Table S16

Table S18. Parameters we use in our evaluation for each tool and dataset in read mapping.

Tool	Dataset	Parameters
BLEND	*CHM13 (HiFi)*	-ax map-hifi -t 32 –secondary=no
BLEND	*HG002 (HiFi)*	-ax map-hifi -t 32 –secondary=no
BLEND	*D. ananassae (HiFi)*	-ax map-hifi -t 32 –secondary=no
BLEND	*E. coli (HiFi)*	-ax map-hifi -t 32 –secondary=no
BLEND	*CHM13 (ONT)*	-ax map-ont -t 32 –secondary=no
BLEND	*Yeast (PacBio)*	-ax map-pb -t 32 –secondary=no
BLEND	*Yeast (ONT)*	-ax map-ont -t 32 –secondary=no
BLEND	*Yeast (Illumina)*	-ax sr -t 32
BLEND	*E. coli (PacBio)*	-ax map-pb -t 32 –secondary=no
minimap2	*CHM13 (HiFi)*	-ax map-hifi -t 32 –secondary=no
minimap2	*HG002 (HiFi)*	-ax map-hifi -t 32 –secondary=no
minimap2	*D. ananassae (HiFi)*	-ax map-hifi -t 32 –secondary=no
minimap2	*E. coli (HiFi)*	-ax map-hifi -t 32 –secondary=no
minimap2	*CHM13 (ONT)*	-ax map-ont -t 32 –secondary=no
minimap2	*Yeast (PacBio)*	-ax map-pb -t 32 –secondary=no
minimap2	*Yeast (ONT)*	-ax map-ont -t 32 –secondary=no
minimap2	*Yeast (Illumina)*	-ax sr -t 32
minimap2	*E. coli (PacBio)*	-ax map-pb -t 32 –secondary=no
Winnowmap2	*CHM13 (HiFi)*	meryl count k=15<br>meryl print greater-than distinct=0.9998<br>-ax map-pb -t 32
Winnowmap2	*HG002 (HiFi)*	meryl count k=15<br>meryl print greater-than distinct=0.9998<br>-ax map-pb -t 32
Winnowmap2	*D. ananassae (HiFi)*	meryl count k=15<br>meryl print greater-than distinct=0.9998<br>-ax map-pb -t 32
Winnowmap2	*E. coli (HiFi)*	meryl count k=15<br>meryl print greater-than distinct=0.9998<br>-ax map-pb -t 32
Winnowmap2	*CHM13 (ONT)*	meryl count k=15<br>meryl print greater-than distinct=0.9998<br>-ax map-ont -t 32
Winnowmap2	*Yeast (PacBio)*	meryl count k=15<br>meryl print greater-than distinct=0.9998<br>-ax map-pb-clr -t 32
Winnowmap2	*Yeast (ONT)*	meryl count k=15<br>meryl print greater-than distinct=0.9998<br>-ax map-ont -t 32
Winnowmap2	*E. coli (PacBio)*	meryl count k=15<br>meryl print greater-than distinct=0.9998<br>-ax map-pb-clr -t 32
LRA	*CHM13 (HiFi)*	align -CCS -t 32 -p s
LRA	*HG002 (HiFi)*	align -CCS -t 32 -p s
LRA	*D. ananassae (HiFi)*	align -CCS -t 32 -p s
LRA	*E. coli (HiFi)*	align -CCS -t 32 -p s
LRA	*CHM13 (ONT)*	align -ONT -t 32 -p s
LRA	*Yeast (PacBio)*	align -CLR -t 32 -p s
LRA	*Yeast (ONT)*	align -ONT -t 32 -p s
LRA	*E. coli (PacBio)*	align -CLR -t 32 -p s
S-conLSH	*CHM13 (HiFi)*	–threads 32 –align 1
S-conLSH	*E. coli (HiFi)*	–threads 32 –align 1
S-conLSH	*CHM13 (ONT)*	–threads 32 –align 1
S-conLSH	*Yeast (PacBio)*	–threads 32 –align 1
S-conLSH	*Yeast (ONT)*	–threads 32 –align 1
S-conLSH	*E. coli (PacBio)*	–threads 32 –align 1
Strobealign	*Yeast (Illumina)*	-t 32

* For the definitions of the parameters we use in BLEND, please see Supplementary Table S16

Table S19. Versions of each tool.

Tool	Version	GitHub or Conda Link to the Version
BLEND	1.0	https://github.com/CMU-SAFARI/BLEND
minimap2	2.24	https://github.com/lh3/minimap2/releases/tag/v2.24
MHAP	2.1.3	https://anaconda.org/bioconda/mhap/2.1.3/download/noarch/mhap-2.1.3-hdfd78af_1.tar.bz2
LRA	1.3.2	https://anaconda.org/bioconda/lra/1.3.2/download/linux-64/lra-1.3.2-ha140323_0.tar.bz2
Winnowmap2	2.03	https://anaconda.org/bioconda/Winnowmap/2.03/download/linux-64/Winnowmap2-2.03-h2e03b76_0.tar.bz2
S-conLSH	2.0	https://github.com/anganachakraborty/S-conLSH-2.0/tree/292fbe0405f10b3ab63fc3a86cba2807597b582e
Strobealign	0.7.1	https://anaconda.org/bioconda/strobealign/0.7.1/download/linux-64/strobealign-0.7.1-hd03093a_1.tar.bz2

Supplementary References

1. M. S. Charikar, “Similarity Estimation Techniques from Rounding Algorithms,” in *Proceedings of the Thiry-Fourth Annual ACM Symposium on Theory of Computing*, ser. STOC ’02. New York, NY, USA: Association for Computing Machinery, 2002.
2. G. S. Manku, A. Jain, and A. Das Sarma, “Detecting Near-Duplicates for Web Crawling,” in *Proceedings of the 16th International Conference on World Wide Web*, ser. WWW ’07. New York, NY, USA: Association for Computing Machinery, 2007.
3. E. S. Tvedte, M. Gasser, B. C. Sparklin, J. Michalski, C. E. Hjelmen, J. S. Johnston, X. Zhao, R. Bromley, L. J. Tallon, L. Sadzewicz, D. A. Rasko, and J. C. Dunning Hotopp, “Comparison of long-read sequencing technologies in interrogating bacteria and fly genomes,” *G3 Genes|Genomes|Genetics*, vol. 11, Jun. 2021.
4. B. Langmead, “Aligning Short Sequencing Reads with Bowtie,” *Current Protocols in Bioinformatics*, vol. 32, Dec. 2010.
5. F. Ramírez, D. P. Ryan, B. Grüning, V. Bhardwaj, F. Kilpert, A. S. Richter, S. Heyne, F. Dündar, and T. Manke, “deepTools2: a next generation web server for deep-sequencing data analysis,” *Nucleic Acids Research*, vol. 44, Jul. 2016.
6. K. Berlin, S. Koren, C.-S. Chin, J. P. Drake, J. M. Landolin, and A. M. Phillippy, “Assembling large genomes with single-molecule sequencing and locality-sensitive hashing,” *Nature Biotechnology*, vol. 33, Jun. 2015.
7. S. Nurk, B. P. Walenz, A. Rhie, M. R. Vollger, G. A. Logsdon, R. Grothe, K. H. Miga, E. E. Eichler, A. M. Phillippy, and S. Koren, “HiCanu: accurate assembly of segmental duplications, satellites, and allelic variants from high-fidelity long reads,” *bioRxiv*, Jan. 2020.
8. H. Li, “Minimap2: pairwise alignment for nucleotide sequences,” *Bioinformatics*, vol. 34, Sep. 2018.
9. J. Ren and M. J. P. Chaisson, “lra: A long read aligner for sequences and contigs,” *PLOS Computational Biology*, vol. 17, Jun. 2021.
10. C. Jain, A. Rhie, N. F. Hansen, S. Koren, and A. M. Phillippy, “Long-read mapping to repetitive reference sequences using Winnowmap2,” *Nature Methods*, Apr. 2022.
11. C. Jain, A. Rhie, H. Zhang, C. Chu, B. P. Walenz, S. Koren, and A. M. Phillippy, “Weighted minimizer sampling improves long read mapping,” *Bioinformatics*, vol. 36, Jul. 2020.
12. A. Chakraborty and S. Bandyopadhyay, “conLSH: Context based Locality Sensitive Hashing for mapping of noisy SMRT reads,” *Computational Biology and Chemistry*, vol. 85, Apr. 2020.
13. A. Chakraborty, B. Morgenstern, and S. Bandyopadhyay, “S-conLSH: alignment-free gapped mapping of noisy long reads,” *BMC Bioinformatics*, vol. 22, Feb. 2021.
14. K. Sahlin, “Flexible seed size enables ultra-fast and accurate read alignment,” *bioRxiv*, Jan. 2022.
